# Molecular imaging and deep learning analysis of uMUC1 expression in response to chemotherapy in an orthotopic model of ovarian cancer

**DOI:** 10.1038/s41598-020-71890-2

**Published:** 2020-09-10

**Authors:** Hongwei Zhao, Hasaan Hayat, Xiaohong Ma, Daguang Fan, Ping Wang, Anna Moore

**Affiliations:** 1 Precision Health Program, Michigan State University, East Lansing, MI 48823 USA; 2 Department of Radiology, College of Human Medicine, Michigan State University, 766 Service Road, Rm. 2022, East Lansing, MI 48823 USA; 3 Shanxi Medical University, Taiyuan, 030001 Shanxi China; 4 Department of Gynecologic Oncology, Shanxi Provincial Cancer Hospital, Taiyuan, 030013 Shanxi China; 5 Lyman Briggs College, Michigan State University, East Lansing, MI 48823 USA; 6 Department of Radiology, National Cancer Center/Cancer Hospital, Chinese Academy of Medical Sciences and Peking Union Medical College, Beijing, 100021 China; 7 Department of General Surgery, Shanxi People’s Hospital, Taiyuan, 030012 Shanxi China

**Keywords:** Nanoparticles, Cancer imaging, Machine learning, Image processing

## Abstract

Artificial Intelligence (AI) algorithms including deep learning have recently demonstrated remarkable progress in image-recognition tasks. Here, we utilized AI for monitoring the expression of underglycosylated mucin 1 (uMUC1) tumor antigen, a biomarker for ovarian cancer progression and response to therapy, using contrast-enhanced in vivo imaging. This was done using a dual-modal (magnetic resonance and near infrared optical imaging) uMUC1-specific probe (termed MN-EPPT) consisted of iron-oxide magnetic nanoparticles (MN) conjugated to a uMUC1-specific peptide (EPPT) and labeled with a near-infrared fluorescent dye, Cy5.5. In vitro studies performed in uMUC1-expressing human ovarian cancer cell line SKOV3/Luc and control uMUC1^low^ ES-2 cells showed preferential uptake on the probe by the high expressor (n = 3, *p* < .05). A decrease in MN-EPPT uptake by SKOV3/Luc cells in vitro due to uMUC1 downregulation after docetaxel therapy was paralleled by in vivo imaging studies that showed a reduction in probe accumulation in the docetaxel treated group (n = 5, *p* < .05). The imaging data were analyzed using deep learning-enabled segmentation and quantification of the tumor region of interest (ROI) from raw input MRI sequences by applying AI algorithms including a blend of Convolutional Neural Networks (CNN) and Fully Connected Neural Networks. We believe that the algorithms used in this study have the potential to improve studying and monitoring cancer progression, amongst other diseases.

## Introduction

In several recent years Artificial Intelligence (AI) algorithms including such as deep learning have demonstrated remarkable progress in image-recognition tasks^1^. These advances are critical for developing clinically translatable tools and systems for rapid quantification and analysis of tumor lesions in order to deliver patient relief and assist doctors in patient management^2,3^. As basic and clinical research continues to advance molecular imaging probes for early cancer detection and monitoring response to therapy, development of AI tools becomes even more important for precise segmentation and quantification of tumor lesions.

**Figure 1:**
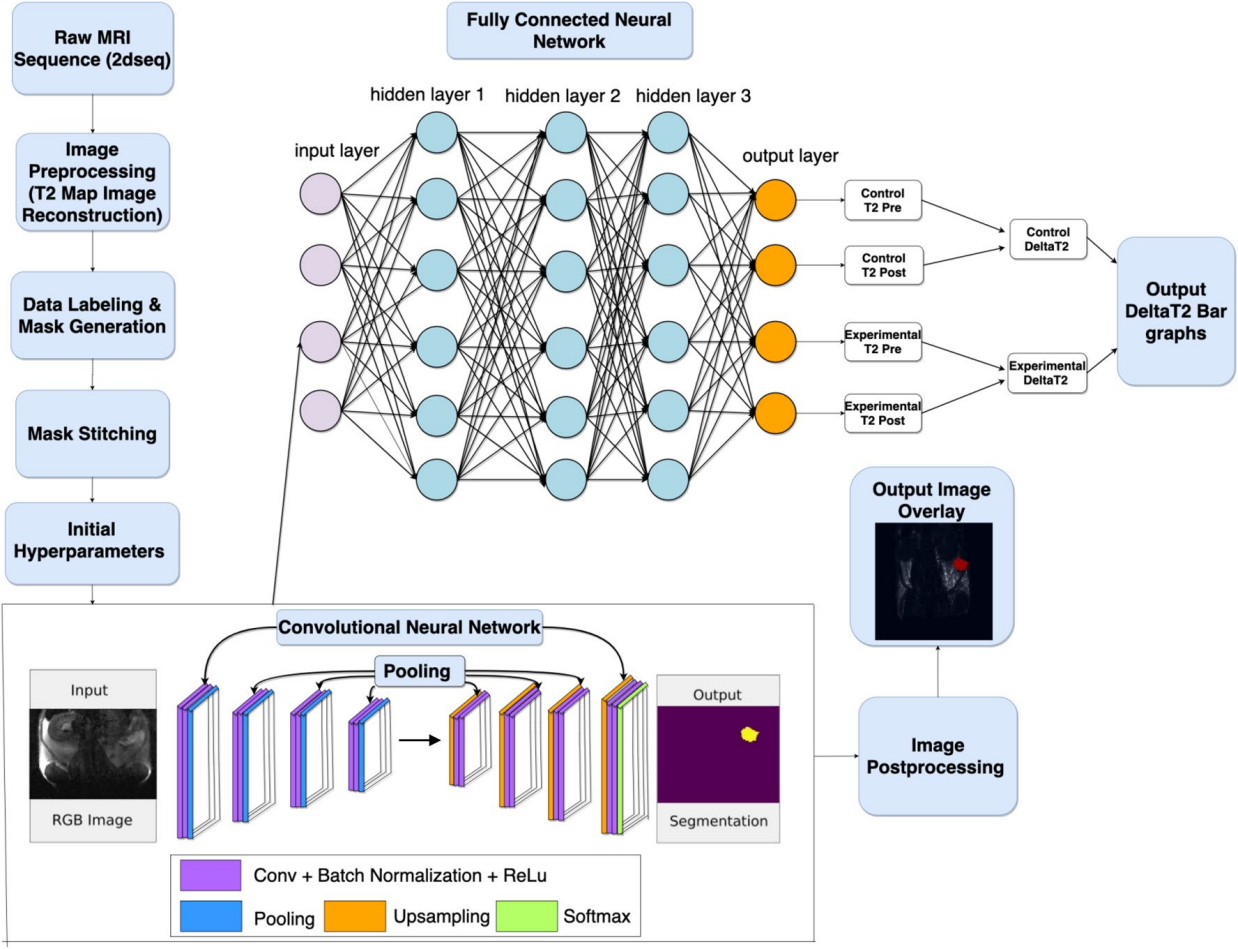
Scheme of deep learning algorithm and associated preprocessing and postprocessing steps for image segmentation and analysis. Image preprocessing of raw MR images consisted of image reconstruction and data mask generation with MATLAB and PyTorch libraries. A Convolutional Neural Network (CNN) was built with TensorFlow and used for image segmentation. A fully connected neural network was employed for analysis of ROI values and calculation of the deltaT2 values.

We have previously described the application of a molecular imaging probe for magnetic resonance imaging (MRI)^4^ targeting the underglycosyslated mucin-1 (uMUC1) antigen^5^. This antigen is overexpressed on almost all human epithelial cell adenocarcinomas^6–14^ as well as on non-epithelial cancer cells^15^ and hematological malignancies^16–18^ comprising more than 50% of human cancers^19^. The uMUC1 antigen exhibits aberrant glycosylation and overexpression in tumor cells^20,21^ and represents an attractive biomarker for molecular imaging of cancer. In addition, uMUC1 availability is directly related to tumor response to chemotherapy suggesting that it can serve as an intermediate biomarker for assessment of therapeutic response and prognosis^22,23^. In our previous studies we demonstrated the utility of in vivo MRI of uMUC1 antigen using targeted magnetic nanoparticles (MN) conjugated to uMUC1-specific peptide EPPT (MN-EPPT) for detection and monitoring of therapeutic response in breast, pancreatic and colon cancers^4,24–28^. While we showed that MN-EPPT could be suitable for detection of uMUC1-expressing tumors and for monitoring changes in uMUC1 expression during chemotherapy, delineation of negatively contrasted tissues on MR images represents a challenge. Therefore, in this current study, we attempted to utilize advanced AI algorithms via deep learning for segmentation and extraction of tumors from negatively contrasted MR images and to conduct AI image analysis of uMUC1 expression probed by MN-EPPT in tumors after chemotherapy. Previously, deep learning algorithms employing convolutional neural network (CNN) delivered segmentation of positive contrast MR images of brain tumor lesions^29,30^. However, the inability of previously generated CNN algorithms to provide adequate quantification of imaging data results from the lack of high throughput segmentation and the minimal tumor analysis algorithms that can provide insight on tumor response to chemotherapy and assessment of probe interference. Therefore, in this study we aimed to utilize novel deep learning algorithms to render high throughput segmentation of tumor region of interest (ROI) as well as an in-depth analysis of tumor response to chemotherapy using an MN-EPPT contrast agent targeting the uMUC1 biomarker. Here we applied these algorithms in an orthotopic murine model of ovarian cancer but given a wide relevance of uMUC1 to human cancers, this approach can be applied for other human malignancies (Fig. 1).

## Results

### MN-EPPT uptake in ovarian cancer cell lines

**Figure 2:**
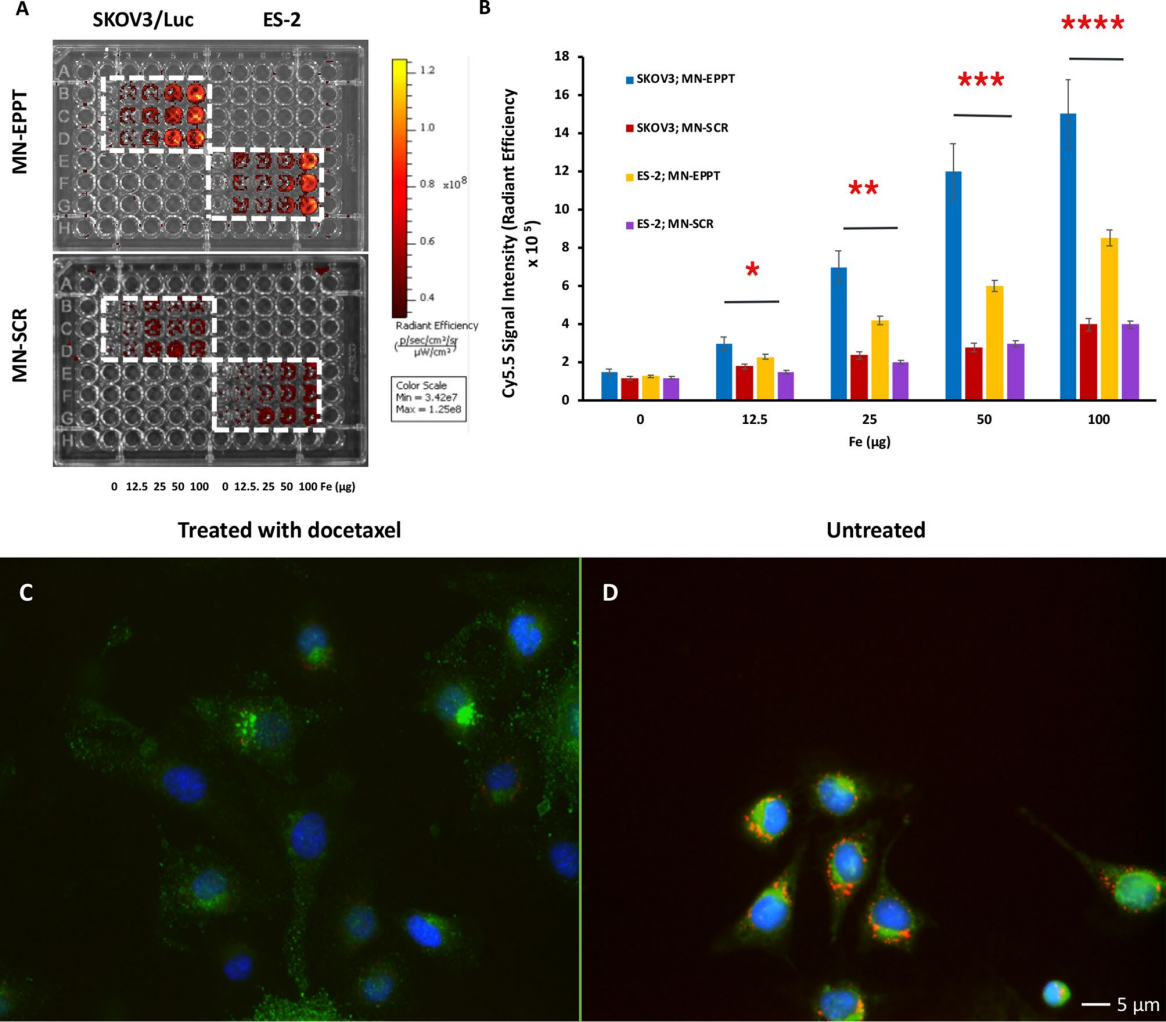
(**A**) In vitro cell binding assay testing relative accumulation of MN-EPPT or MN-SCR probes in SKOV3/Luc and ES2 cell lines (n = 3). (**B**) Quantitative analysis of cell binding assays showed preferential, concentration dependent uptake of MN-EPPT probe by SKOV3/Luc cells compared to scrambled control probe. ES-2 cells exhibited significantly lower uptake of MN-EPPT probe. (*p value < .05 for student T-test). (**C**) Fluorescence microscopy of SKOV3/Luc cells after treatment with docetaxel. (**D**) Untreated SKOV3/Luc cells. Changes in the expression of the uMUC1 tumor antigen and a reduction in relative MN-EPPT probe accumulation are apparent in the docetaxel-treated cells (green—uMUC1; red—Cy5.5 conjugated to the nanoparticles; blue—DAPI, nuclei; magnification bar = 5 µm).

In order to measure relative accumulation of MN-EPPT and scrambled control probe MN-SCR in the cells, uMUC1-expressing SKOV3/Luc and uMUC1^low^ control ES-2 ovarian tumor cells were incubated with these probes. As shown in Fig. 2A,B, SKOV3/Luc cells exhibited significantly higher concentration dependent uptake of uMUC1-specific MN-EPPT probe compared to the scrambled MN-SCR control probe (*p* < 0 0.05; n = 3). ES-2 tumor cells incubated with both experimental and control probes showed significantly lower uptake of MN-EPPT probe and negligible uptake of MN-SCR probe (*p* < 0.05, n = 3). Since uMUC1 expression is present in ES-2 although at negligent low level^31^, the low uptake of MN-EPPT is expected. There was no difference in uptake of the scrambled probe between the cell lines.

### Fluorescence microscopy of docetaxel treated cells

To investigate whether changes in uMUC1 expression in response to chemotherapy can be monitored by the probe, we treated SKOV3/Luc cells with docetaxel and probed them with MN-EPPT. Fluorescence microscopy showed the apparent reduction in the uMUC1 tumor antigen expression as probed by MN-EPPT. The reduced probe accumulation as a result of the loss of the uMUC1 epitope targeted by MN-EPPT is evident in docetaxel treated cells (Fig. 2C) compared to untreated cells (Fig. 2D). Corrected Total Cell Fluorescence (CTCF) quantification in the uMUC1 channel (Supplementary Fig. 2A) and Cy5.5 channel (Supplementary Fig. 2B) indicated a statistically significant difference in relative uMUC1 expression and MN-EPPT probe accumulation between cells treated with docetaxel and untreated cells (*p* < 0.05). Cells treated with docetaxel showed reduced uMUC1 expression and lower probe accumulation than untreated cells. These results indicate that accumulation of the probe in tumor cells treated with chemotherapy could be reduced, which is an important finding for devising strategies for monitoring tumor response to chemotherapy.

### MN-EPPT-enhanced MRI and optical imaging

**Figure 3:**
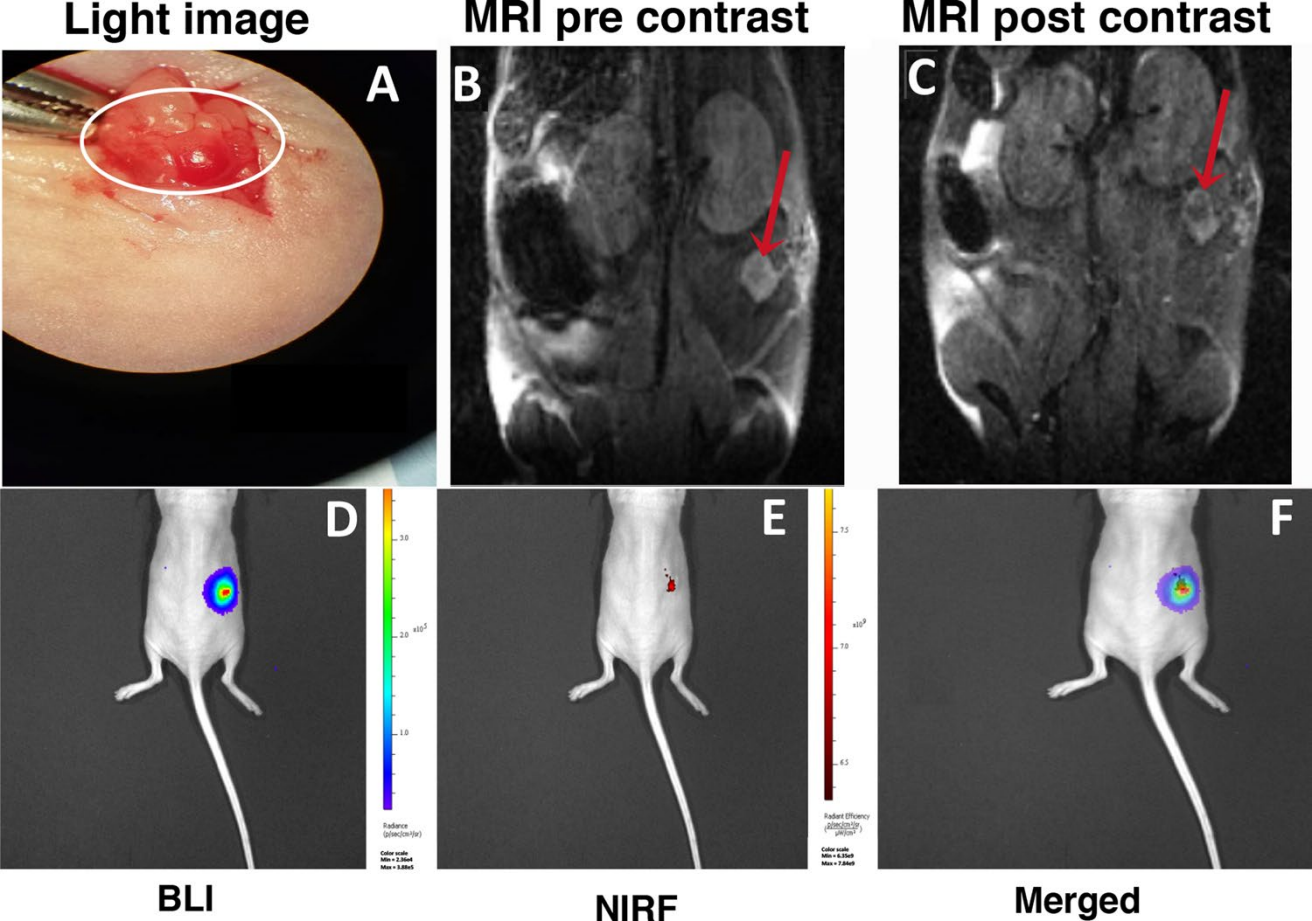
(**A**) Light image of tumor lesions 7 days post orthotopic transplantation of tumor cells in the left ovary. (**B**) T2WI pre-contrast MR image of a mouse with ovarian tumor. (**C**) T2WI MR image 24 h after injection of MN-EPPT. (**D**) Bioluminescence imaging (BLI) of SKOV/Luc tumor. (**E**) Near infrared fluorescence imaging (NIRF) of MN-EPPT probe accumulation in ovarian tumors. (**F**) Merged BLI and NIRF images show co-localization of the corresponding signals.

Accumulation of the probes in SKOV3/Luc ovarian tumors was monitored by MR and optical imaging. A light image of the implanted orthotopic tumor is shown in Fig. 3A. MR imaging showed a decrease in signal intensity on post-contrast T2W MR images 24 h after MN-EPPT injection (Fig. 3B,C). Optical imaging performed immediately after the MR session demonstrated co-localization of the bioluminescence signal from the tumor (Fig. 3D) with the near infrared signal originating from the Cy5.5 dye conjugated to the nanoparticles (Fig. 3E). These results confirmed accumulation of MN-EPPT in orthotopic ovarian tumors (Fig. 3F), which we further exploited for monitoring tumor response to chemotherapy.

### Biodistribution of MN-EPPT probe in mice treated with docetaxel

**Figure 4:**
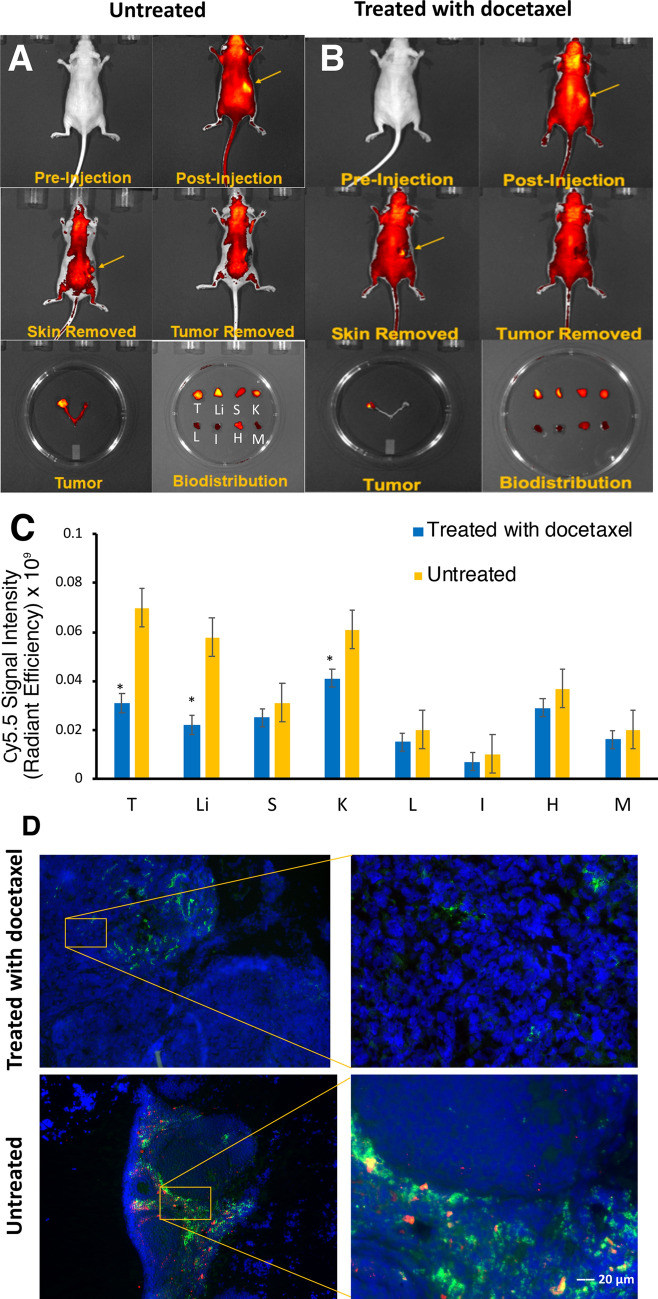
Biodistribution of MN-EPPT probe in tumor bearing mice treated with docetaxel (n = 5) or left untreated (n = 4). (**A**,**B**) Top row: Near infrared optical imaging of mice from both groups before and after injection of MN-EPPT. (**A**,**B**) Middle row showing mice with removed skin for confirmation of tumor localization and probe accumulation (left image). Removal of the tumor results in disappearance of the NIRF signal (right image). (**A**,**B**) Bottom row: excised ovaries indicated tumoral accumulations of MN-EPPT (left image). Biodistribution of the probe in major organs (right image). (**C**) Quantitation of biodistribution shows reduced uptake of MN-EPPT by the tumors treated with docetaxel. (**D**) Immunofluorescence staining of ovarian tumor tissue sections from animals treated with docetaxel (top row, low and high res) and immunofluorescence staining of ovarian tumor tissue sections from untreated animals (bottom row, low and high res). Green—uMUC1, red—MN-EPPT (Cy5.5 on MN), blue—DAPI nuclear stain. Magnification bar = 20 µm.

To quantify changes in uMUC1 expression in response to chemotherapy in vivo we performed ex vivo biodistribution studies. MN-EPPT probe was injected in the docetaxel treated animals (n = 5) or in the saline treated control group (n = 4). Localization of the probe in the tumors 24 h post injection is shown in Fig. 4A,B (top row). Confirmation of probe localization was obtained after skin removal which showed an obvious signal from the tumor. This signal disappeared after the tumor was excised (Fig. 4A,B, middle row). The presence of the signal in the tumors in the left ovary was also confirmed on excised organs (Fig. 4A,B, bottom row). Biodistribution studies demonstrated that while we observed probe accumulation in the tumors in both experimental and control groups, mice treated with chemotherapy exhibited lower probe accumulation and therefore reduced signal intensity from their tumors (Fig. 4C). Furthermore, several organs in docetaxel treated animals such as the liver and kidney accumulated lower levels of MN-EPPT, a phenomenon which requires future examination. It is apparent from these experiments that mice that did not receive chemotherapy likely maintained higher levels of uMUC1 antigen expression as a result of continuous tumor growth and further invasion, resulting in greater accumulation of MN-EPPT. Downregulation of uMUC1 antigen after chemotherapy resulted in reduced probe accumulation as evident from the reduction in signal intensity in treated tumors (*p* < 0.05; Fig. 4C). To assess the level of uMUC1 expression after chemotherapy we next performed qRT-PCR of tumor tissues from experimental and control mice. As indicated in the Supplementary Fig. 1, a significant downregulation of uMUC1 expression at the RNA level was observed in mice treated with docetaxel compared to the untreated control group. Fluorescence microscopy performed at the end of the study confirmed a significant reduction in uMUC1 expression at the protein level in tumors treated with docetaxel compared to untreated tumors (Fig. 4D). CTCF quantification of fluorescence histology of tumor lesions of treated and untreated mice indicated a statistically significant reduction in staining for uMUC1 and for Cy5.5 labeled probe in mice treated with docetaxel in comparison to untreated mice (*p* < 0.05; Supplementary Fig. 3). This reduction paralleled lower accumulation of MN-EPPT probe in treated animals observed by imaging.

### RECIST classification and MR image analysis by deep learning algorithm

**Figure 5:**
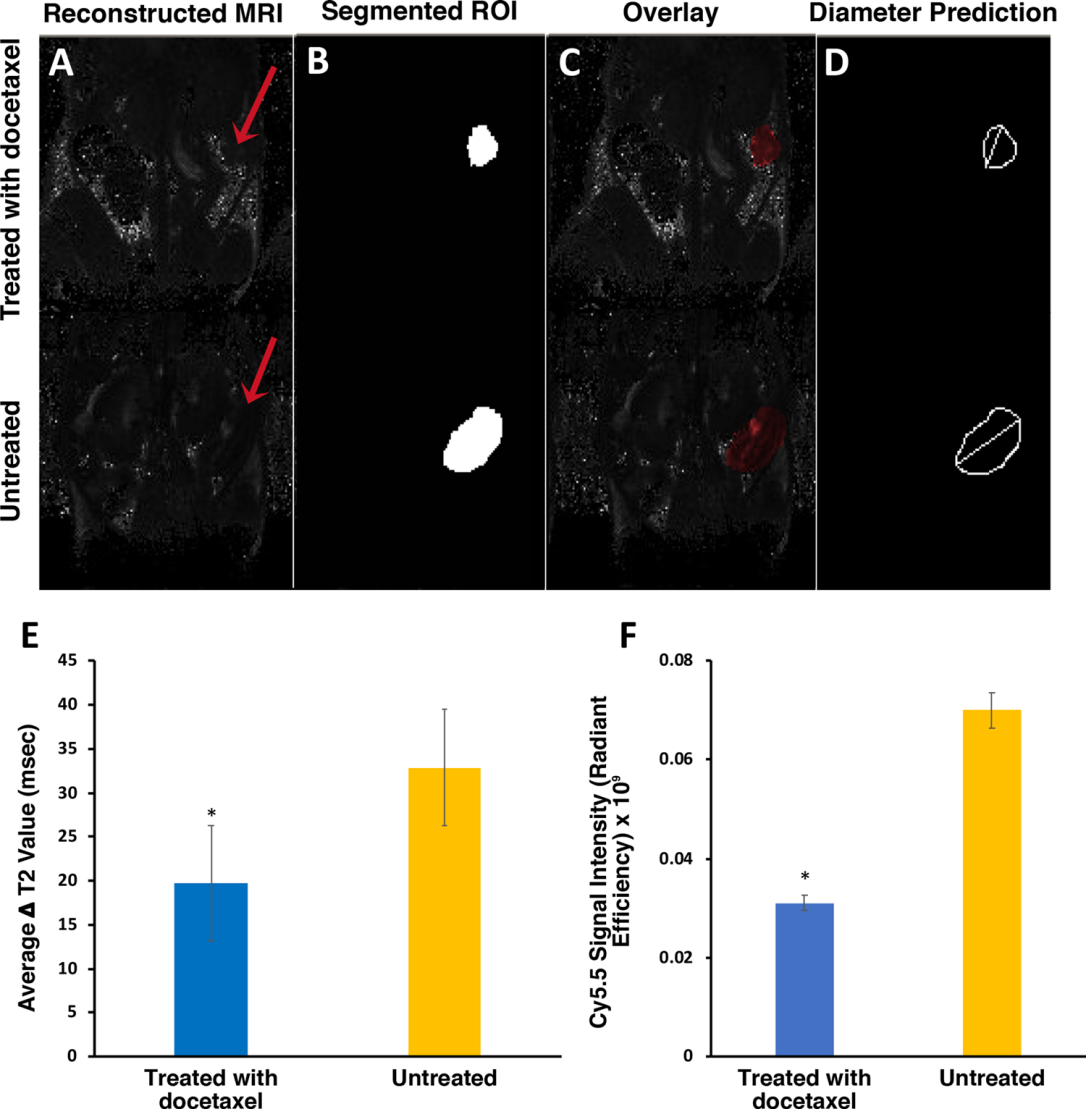
Deep learning algorithm tumor segmentation and analysis. (**A**) Original reconstructed T2 Map of MR images. (**B**) Segmented ROI from deep learning algorithm. (**C**) Overlay of segmentation result on original reconstructed MR image. (**D**) Diameter reading from the segmented ROI. (**E**) Average deltaT2 values of tumors in treated and untreated groups obtained from deep learning algorithm. (**F**) MN-EPPT probe accumulation as a function of Cy5.5 signal intensity (radiant efficiency) from optical images of docetaxel-treated and untreated groups.

The deep learning algorithm was able to calculate diameter readings for the segmented tumor ROI from experimental (treated) and control (untreated) groups (Fig. 5A–D). From the segmented ROI extracted by the algorithm, a classification model was set up in order to allow the algorithm to measure the change in tumor diameter over time and observe tumor response to chemotherapy. From the predictions made by the algorithm, we made a conclusion that two of the mice exhibited a progressive response (PR) to chemotherapy, indicating a greater than 30% reduction in tumor size over the course of chemotherapy. Two additional mice exhibited no significant change in tumor diameter over the course of chemotherapy, and were classified into a steady disease (SD) group. One mouse showed a significant increase in tumor diameter over the course of treatment and was classified as a progressive disease (PD). After segmenting the tumor ROIs, the algorithm was then able to predict the deltaT2 values between the experimental and control groups (Fig. 5E). The delta T2 values indicated that there was a significantly lower change in T2 experienced by the treated group as compared to the untreated control (*p* < 0.05). Near infrared optical imaging confirmed these results showing the higher relative probe accumulation in untreated mice as compared to those that received chemotherapy (Fig. 5F).

### Intraclass correlation coefficient (ICC) validation

**Figure 6:**
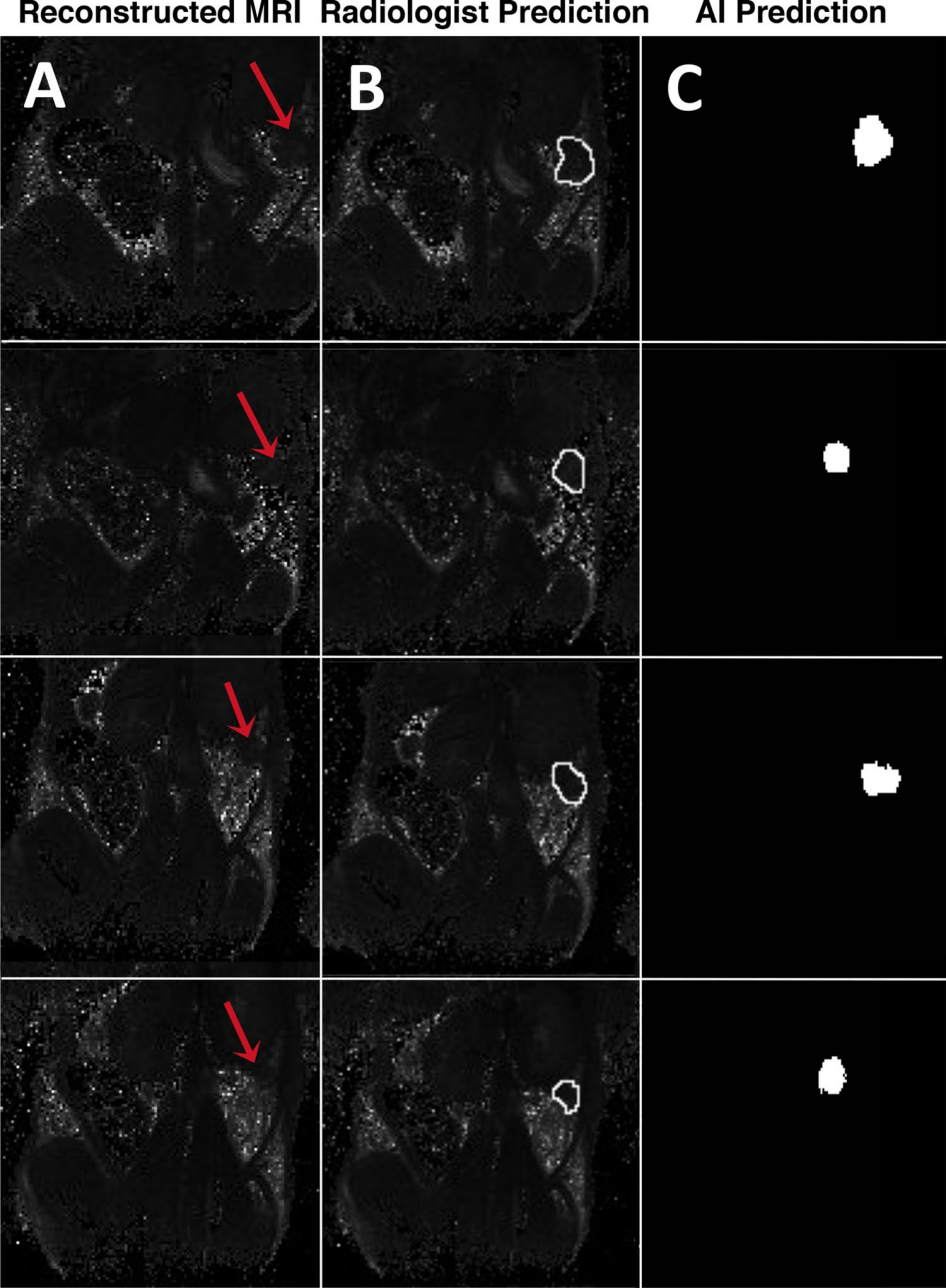
Representative visual example of MR images used for ICC cross validation between the board-certified radiologist prediction and the deep learning algorithm prediction. (**A**) Original reconstructed MR image. (**B**) Prediction of tumor ROI labeled by a board-certified radiologist. (**C**) Prediction of tumor ROI by the deep learning algorithm segmentation.

Figure 6 depicts a few of the several tumor ROI predictions by the two raters: the radiologist and the deep learning neural network. Reconstructed T2 maps from docetaxel treated groups are presented in Fig. 6A, as well as ROI of both treated and untreated groups predicted by raters (Fig. 6B,C). The level of agreement amongst their predictions was calculated using the ICC cross validation in SPSS statistical software.

Table 1A and B summarize Intraclass Correlation Coefficient (ICC) between the predictions of the two raters: the board-certified radiologist and the deep learning algorithm. Ordinarily, acceptable ICC scores range between 0.7 and 0.9, with the score greater than 0.9 considered excellent. It can be seen from Table 1A and B that there is a greater degree of correlation and agreement amongst the predictions of tumor ROI sum (size) than the ICC for tumor circularity between the two raters, as indicated by the single measure values 0.890 and 0.856 for tumor size and shape, respectively. This is likely due to the degree of error that is introduced when contouring the tumor manually, frequently leading to minor discrepancies in the shape of the tumor prediction, which is often remediated by the prediction of tumor size between the two raters. That is, the exact shape of the tumor may differ amongst radiologists and the artificially intelligent neural network, however, the ROI size and total number of pixels remains relatively constant. The latter value is of greater importance than tumor shape when preforming tumor analysis such as calculating deltaT2 values between different images and measuring relative probe accumulation in response to chemotherapy. Therefore, it can be validated from the ICC score that there is a high degree of interrater reliability between the AI and board-certified radiologist in regard to segmentation of the proper tumor ROI from a negative contrast pixel field of the reconstructed MR image.

**Table 1:** Interclass correlation coefficient of (**A**) tumor ROI sum (size), (**B**) tumor circularity.

	Interclass correlation^a^	95% confidence interval		F test with true value 0			
		Lower bound	Upper bound	Value	df1	df2	Sig
**(A)**							
Single measures	0.890^b^	0.797	0.942	18.067	37	37	0.000
Average measures	0.942^c^	0.887	0.970	18.067	37	37	0.000
**(B)**							
Single measures	0.856^b^	0.729	0.925	13.748	34	34	0.000
Average measures	0.922^c^	0.843	0.961	13.748	34	34	0.000

Two-way mixed effect model where people effects are random and measures effects are fixed.

^a^Type A interclass correlation coefficient using an absolute agreement definition.

^b^The estimator is the same, whether the interaction effect is present or not.

^c^This estimate is computed assuming the interaction effect is absent, because it is not estimable otherwise.

## Discussion

Changes in tumor antigen expression following chemotherapy can be indicative of tumor response to treatment. In case of uMUC1, it was shown that its downregulation following therapeutic intervention decreases the invasive potential of cancer cells, reduces metastatic burden, and improves survival^32–34^. In this study we investigated the response to chemotherapy (docetaxel) in ovarian cancer by monitoring uMUC1 downregulation probed by MN-EPPT imaging probe. In order to analyze tumor size and measure the response to treatment, MR images before the first round of chemotherapy and after the last round of treatment were evaluated. From a thorough analysis of the Feret’s diameters in experimental subjects, mice were categorized according to RECIST benchmarks and revealed SD, PR, and PD categories. In saline treated control groups, tumor lesions grew to nearly twice their baseline size over the course of 4 weeks. Because SD and PR are indicative of successful cancer treatment, it is apparent that the tumor response to chemotherapy and uMUC1 antigen expression can be interpreted from MN-EPPT-enhanced MR imaging. MRI results and AI analysis indicated a reduction in antigen expression and probe accumulation in docetaxel-treated mice, and therefore a decrease in deltaT2 obtained from T2 maps was observed. Furthermore, as there is no signal interference in the tumor region before injection of the probe, the change in the T2 value over the region can be used to calculate the deltaT2 using AI (Deep Learning) based analysis. The results from the deep learning algorithm indicated a reduced deltaT2 value in docetaxel-treated mice as compared to untreated mice (Fig. 5). This indicates the apparent downregulation of the uMUC1 tumor antigen confirmed by RT-PCR and fluorescence microscopy and reduced probe accumulation in the treated tumors as a consequence. Additionally, ICC validation of the deep learning algorithm determined that the model was preforming with a high degree of accuracy in comparison to board certified radiologists, and this permits the validity of the metrics measured and analyzed by the neural network (Table 1A and B). Our results indicate that ovarian cancer can be monitored via MN-EPPT-enhanced MRI, and its progression/response to treatment may undergo analysis via an AI algorithm such as that employed in this study with a high degree of accuracy.

There are limitations of our current studies. We have not correlated the data from T2 map and algorithm prediction to the real tumor volume. The main function of the algorithm we developed was to calculate delta T2 to assess probe accumulation (and therefore, biomarker expression) in response to chemotherapy. We only used the diameter from one dimension since the T2 map used for analysis was a coronal view and therefore the Z dimension was not taken into consideration when estimating tumor size with this algorithm. This is an inherent limitation of extending our algorithm to RECIST classification by measuring changes in diameter across experimental and control groups, however, our algorithm-segmented ROIs are accurate to a greater degree compared to board-certified radiologists, as per our ICC scores. For the ROI measurements, we used the Feret’s Diameter, which is the longest diameter present in a single slice. We applied this method to an entire T2map of 15 slices as the basis for measuring the comparison between different time points (pre-chemo and post-chemo). Development of an algorithm for three-dimensional tumor volume prediction will be the focus of our future studies.

The paradigm of AI unlocks a great deal of analytical potential that can be harnessed in the field of precision medicine. With accurate, high throughput analysis of tumor lesions and probe accumulation obtained from MR images, clinicians and researchers may be able to better and faster monitor treatment outcomes without the presence of a selection bias when evaluating tumor ROI. This may permit real-time monitoring of tumor response to chemotherapy, and can lead to better-informed decisions in the clinic when determining the course of future treatment and prognosis. The fact that AI has the ability to perform with a relatively high degree of agreement with board certified radiologists underscores its potential for use in studying and monitoring cancer progression, amongst other diseases. Deep learning, the most contemporary form of AI employed in this study, renders a paradigm shift in the current approach to cancer diagnosis, prognosis, and treatment, and stands at the forefront of advancements in the future of medicine.

## Methods

### Synthesis of the imaging probes

Iron-oxide based dextran-coated magnetic nanoparticles (MN) were synthesized as described previously^27^. Experimental probe (MN-EPPT) was synthesized by conjugating iron oxide nanoparticles to the EPPT peptide (C-AHA-Y-C(ACM)-A-R-E-P-P-T-R-T-F-A-Y-W-G-K)^27^. Near infrared optical dye (Cy5.5) was conjugated to the nanoparticles for fluorescence microscopy and correlative near infrared optical imaging. Briefly, Cy5.5 mono-reactive NHS ester (GE Healthcare) was dissolved in DMSO and incubated with MN in 20 mM citrate buffer (pH 8.0) overnight followed by purification on Sephadex PD-10 column (GE Healthcare). Cy5.5-labeled MN (MN-Cy5.5) were loaded with hetero-bifunctional crosslinkers, N-γ-Maleimidobutyryl-oxysuccinimide ester (GMBS) followed by conjugation with EPPT peptide modified with a thiol group at its N-terminus (NH2-Cys-(PEG)-Tyr-Cys(acm)-Ala-Arg-Glu-Pro-Pro-Thr-Arg-Thr-Phe-Ala-Tyr-Trp-Gly-Lys-CONH_2_). The number of Cy5.5 molecules per nanoparticle was determined by spectrophotometry. The number of EPPT peptide molecules was quantified using a bicinchoninic acid protein assay (BCA assay, Pierce). Iron concentration was determined spectrophotometrically using Iron Assay kit (Sigma-Aldrich). Loading ratios of Cy5.5 and the EPPT peptide per nanoparticle were 8.7 and 2.3, respectively. The core size of the probe was 22.31 ± 0.54 nm. Control probe (MN-SCR) was synthesized using a scrambled peptide sequence (C-AHA-A-E-G-R-P-T-F-P-T-R-A-Y-W-K) conjugated to MN.

### In vitro cell binding assay and immunocytochemistry

Human luciferase-expressing ovarian cancer cell line SKOV3/Luc (expressing uMUC1 tumor antigen, Cellbiolabs, Inc., catalog number AKR-232) and ES-2 uMUC1^low^ (ATCC Cat# CRL-1978, RRID:CVCL_3509), control cells expressing the lowest amount of uMUC1 among ovarian adenocarcinomas^31^) were used for this study. Cell lines were not specifically tested for authenticity as they were obtained from reliable commercial sources and used within 6 months after receipt or resuscitation of the original passage. Every two months cells were tested for mycoplasma contamination (Hoechst 33342 stain) and maintained as mycoplasma-free culture. Cells were routinely used within the first 10 passages. For assessment of the relative accumulation of experimental (MN-EPPT) and control (MN-SCR) nanoparticle probes within the tumor cells, the SKOV3/Luc and ES-2 cell lines were seeded into 96-well plates and incubated with either MN-EPPT or MN-SCR at 0, 12.5, 25, 50, and 100 μg/ml Fe at 37 °C for 1 h (n = 3). Following the incubation, accumulation of the probes was evaluated by Cy5.5 fluorescence using an IVIS spectrum imaging system (PerkinElmer, Hopkinton, MA), and normalized to total cell protein as measured by BCA protein assay.

In order to evaluate uMUC1 downregulation in response to chemotherapy, SKOV3/Luc cells were incubated with 0.2 μmol/l docetaxel at 37 °C for 24 h. Cells treated with saline solution served as a negative control. Next, cells were incubated with MN-EPPT at 37 °C for 1 h, washed and subjected to fluorescence microscopy. Cells were incubated with anti-mucin 1 rabbit polyclonal antibody (1:100 dilution; Abcam, Cambridge, MA), with a corresponding fluorescently labeled IgG secondary antibody (1:200 dilution; Abcam, Cambridge, MA) for visualization of the uMUC1 tumor antigen. The cells were counterstained with DAPI-containing mounting medium (Vectashield; Vector Laboratories, Inc., Burlingame, CA) for visualization of cell nuclei. Fluorescent images were observed using a Nikon Eclipse 50i fluorescence microscope (Nikon, Melville, NY), equipped with appropriate fluorescence filter sets. Images were analyzed using SPOT 4.0 Advance version software (Diagnostic Instruments, Sterling Heights, MI. Multi-color fluorescent images were split into single channels and converted to grayscale images. Regions of interest (cells with uMUC1 expression and probe accumulation) were selected via the freehand selection tool in ImageJ 1.46r software (NIH). The corrected total cell fluorescence (CTCF, a unit of measurement utilized by ImageJ to quantify specific levels of fluorescence) was calculated using the formula: CTCF = Integrated Density − (total area of selected cell × mean fluorescence of background region). A student T-test was used for statistical evaluation of results, with a *p* value < 0.05 indicating statistical significance.

### Orthotopic murine model of ovarian cancer

All animal experiments were performed in compliance with the National Institutes of Health guide for the care and use of Laboratory animals (NIH Publications No. 8023, revised 1978) and according to guidelines and regulations as described in Protocol 03-18-033-00 approved on 10/10/2018 by Michigan State University Institutional Animal Care and Use Committee. Initial imaging studies were performed in accordance with the protocol 2006-N000065 approved on 4/3/2015 by Massachusetts General Hospital Institutional Animal Care and Use Committee. In order to establish an orthotopic murine model of ovarian cancer, isoflurane anesthetized 8-week-old female nude Athymic L2R1 mice (Jackson Laboratories, Bar Harbor, ME) were inoculated with 1 × 10^6^ of SKOV3Luc cells in the left ovary. Tumor growth/regression was monitored using bioluminescence imaging (IVIS Spectrum, Perkin Elmer, Hopkinton MA). We used four groups of animals: (1) animals treated with chemotherapy and probed with MN-EPPT (n = 5); (2) animals treated with chemotherapy and probed with MN-SCR (n = 3); (3) animals treated with saline solution injections and probed with MN-EPPT (n = 4); and (4) animals treated with saline solution injections and probed with MN-SCR (n = 3).

### Chemotherapy regimen and tumor monitoring

Chemotherapy treatment with docetaxel began on day 7 after initiation of the tumors and consisted of three rounds of intraperitoneal injections of docetaxel in saline solution once a day for 5 consecutive days with a two days break. Control mice received intraperitoneal injections of saline solution. Assessment of tumor response to chemotherapy was done according to the response evaluation criteria in solid tumors (RECIST). A complete response (CR) involves apparent dissolution of the target tumor lesion, while a partial response (PR) indicates a ≥ 30% decrease in the longest diameter of target lesion in comparison with the baseline levels. Progressive disease (PD) constitutes a ≥ 20% increase in the longest diameter of the target tumor lesion in comparison with the recorded baseline, or can be indicated by multiple new tumors. Stable Disease (SD) occurs when neither PR or PD occurs. As determined through RECIST, effective chemotherapy treatment regimens result in CR, PR, and SD, whereas ineffective treatments often result in PD.

### In vivo imaging

To evaluate uMUC1 expression in response to chemotherapy, animals were injected with either MN-EPPT or MN-SCR probes (15 mg Fe/kg) and subjected to MR and near infrared fluorescence optical imaging before and after the first and last rounds of chemotherapy. MR imaging was performed before and 24 h after the probe injection using a 9.4 T Bruker horizontal bore scanner equipped with a rat array cryoprobe coil (Bruker, Billerica, MA). Images were processed by fitting the voxel T2 values to an exponential decay curve within 15 slices (y = A exp(− t/T2) implemented using MATLAB (R2015b, Mathworks, Inc., Natick, MA)) to generate a comprehensive T2 map for segmentation, ROI analysis, and deltaT2 calculations. T2 values were calculated by automatic segmentation of the tumor region of interest (ROI) via a deep learning algorithm consisting of a recurrent Convolutional Neural Network (rCNN) for semantic segmentation of the tumor ROI from negative contrast MR images as described in the next section. Data were then passed through a fully connected neural network for deltaT2 calculations and analysis in order to compare the deltaT2 values of both experimental and control groups. Tumor size was calculated using the Feret’s diameter value (longest tumor diameter).

In vivo near infrared optical imaging was performed immediately after the MRI session using an IVIS Spectrum imaging system. For analysis of probe accumulation in cancer lesions, ROIs were drawn around each tumor using LivingImage 4.5 software (https://www.perkinelmer.com/lab-products-and-services/resources/in-vivo-imaging-software-downloads.html#LivingImage, PerkinElmer, Hopkinton, MA). In order to quantify the ROI, fluorescence emissions were measured as average radiant efficiency, which is the fluorescence emissions normalized to the incident excitation intensity. Mice were sacrificed following the final round of imaging, tumors and other major organs were extracted and imaged ex vivo using an IVIS Spectrum imaging system.

### Ex vivo RT-PCR of tumor tissues and fluorescence microscopy

Tumor tissue RNA was extracted using the RNeasy Mini Kit (Qiagen) with DNase treatment (Invitrogen), and cDNA was synthesized using miScript II RT kit (Qiagen). Real-time PCR reactions were performed with QuantiTect SYBR Green PCR Kits (Qiagen) and analyzed using ∆∆Ct methodology. TBP was used as a normalization gene. Primers (uMUC1gene, forward primer, reverse primer) were purchased from Integrated DNA Technologies. Immunofluorescence staining was performed to evaluate uMUC1 expression levels as well as MN-SCR and MN-EPPT probe accumulation in the tumor tissue samples. Staining was performed on 5 μm thick frozen tissue samples from mouse ovarian tumors. The tissue samples were stained for the uMUC1 antigen using an anti-MUC1 primary antibody (1:100 dilution; Abcam, Cambridge, MA), followed by an FITC-labeled secondary antibody and counterstained with DAPI (Vectashield; Vector Laboratories, Inc., Burlingame, CA). Fluorescent images were examined using a Nikon Eclipse 50i fluorescence microscope, equipped with the appropriate filter sets and analyzed using SPOT 4.0 Advance version software (https://www.spotimaging.com/software/spot-advanced/). CTCF for quantification of ex vivo fluorescence microscopy images was preformed using the methods previously discussed.

### MR imaging analysis using a deep learning algorithm

In an effort to accurately and semantically segment tumor lesions from negative contrast MR images of the orthotopic ovarian tumors and perform automated ROI analysis and probe accumulation analysis, a recurrent Convolutional Neural Network (CNN) was engineered through PyTorch and Tensorflow libraries (Python v3.3). Through analysis of the segmented ROI output from the neural network, probe accumulation within the tumors was measured through pixel intensity calculation in respect to time, calculated for images taken pre-injection and post- injection of the probes. The obtained deltaT2 values allow for a measurement of MN-EPPT and MN-SCR probe accumulation in the tumors and can therefore allow for comparison of relative uMUC1 antigen expression between experimental and control groups. The neural network capable of determining the deltaT2 values was created and a build-train-test model was enforced. Raw 2dseq format of images was preprocessed via MATLAB T2 map-generating software (MathWorks, Natick, MA) as mentioned previously and masks of the tumor contours were generated using LabelMe software (Massachusetts Institute of Technology, Cambridge, MA) for each slice of the T2 map. After mask generation, the masks were stitched and fed into the neural network for training. Once adequate connection weights were established, a new set of unmarked, ambiguous images were input into the neural network for testing. Each T2map was passed through a 15-channel tensor which evaluated each one of the fifteen slices in a T2map individually and sequentially in order to locate and extract the tumor ROI. Once tumor contour was recognized and segmented by the neural network, the segmented ROI was measured as average pixel intensity (total pixel intensity in ROI /total number of pixels in ROI). The ROI was extracted from each slice of the T2 map using the CNN algorithm. Our algorithm uses the function of “dimensionality reduction” and converges onto an ROI that it estimates the true ROI based of the data the algorithm has been trained on. This is a canonical method used in image segmentation via machine learning. We have validated that the segmented ROI from a slice is comparable to that of a board-certified radiologist hand-drawn counterpart via our ICC statistical correlation values. Therefore, after obtaining the ROI from one slice in the T2map of mice before probe injection, the individual voxel values are summed to give us the total pixel intensity of the ROI. This was done for each slice in that T2 map. Once the algorithm has sorted through all of the slices, the pixel sum of each of the individual ROIs from each slice are totaled and averaged to give us the average T2 for that entire T2 map. Once the average T2 value is calculated for that map, we input another image sequence from post-injection image into the algorithm, and the above process to get average T2 value is repeated. Once we have average T2s for both pre- and post-injection images, deltaT2 value for the subject was calculated by subtracting the post-injection mean ROI from the pre-injection mean ROI (mean ROIpre—mean ROIpost = deltaT2). This provides us with the level of probe interference according to T2 value, which is related to antigen expression. The scheme for Deep Learning Algorithm for ovarian tumor segmentation and analysis is presented in Fig. 1.

### Gradient descent loss algorithm for training and ground truth data set

In order to train the CNN to increase accuracy and throughput of segmentation and analysis results, the gradient descent loss algorithm was configured into the model for every iteration of new training images that was fed into the neural network. This deep-learning algorithm allows the neural network to converge at a local minimum when calculating loss^35^. The gradient value calculated by gradient descent loss indicates the current slope of our cost function at the current position; a negative value indicates a decrease in dice loss value, and this is indicative of the model learning. The network calculates cost and therefore dice loss by running the CNN with previous weights and bias, and coordinates its results with a previously established ground truth. Ground truth is the initial training dataset that establishes the primal nodes in the neural network and provides a basis from which the network can compare the current calculated output and calculate cost dice loss. Dice loss is indicative of the number of false positives over the true number of positives. As dice loss decreases to converge at the local minimum on the parabola, an apparent increase in the machines accuracy and reliability is often observed^36^. The cost function in respect to weight (m) and bias (b) is shown below, and the derived gradient functions implemented in Tensorflow, using fast.ai and PyTorch libraries (Python library), are listed below as well^37^.

Cost Function:

$$f\left( {m,b} \right) = \frac{1}{N}\mathop \sum \limits_{i = 1}^{n} (y_{i} - \left( {mx_{i} + b} \right))^{2}$$

Gradient Function:

$$f^{\prime}\left( {m,b} \right) = \left[ {\begin{array}{*{20}c} {\frac{df}{{dm}}} \\ {\frac{df}{{db}}} \\ \end{array} } \right] = \left[ {\begin{array}{*{20}c} {\frac{1}{N}\sum - 2x_{i} \left( {y_{i} - \left( {mx_{i} + b} \right)} \right)} \\ {\frac{1}{N}\sum - 2\left( {y_{i} - \left( {mx_{i} + b} \right)} \right)} \\ \end{array} } \right]$$

In order to change the outcome and direction of training, various hyperparameters including learning rate (lr), batch size, and weight distribution (wd) could be manipulated from iteration to iteration. The initial hyperparameters for this neural network were as follows: lr = 4e^−3^, batch size = 8, and wd = 1e^−7^. In order to effectively exploit the gradient descent loss algorithm, images for initial training (ground truth) were analyzed by clinical radiologists for representativeness of the ovarian tumors and relative accumulation of the probe causing changes in T2. About 600 images from 40 T2 maps reconstructed from MATLAB software were selected for ground truth, with an equal distribution of MR images from both treated and untreated groups (control and experimental). Subsequent training rounds included up to 400 additional images on which the gradient descent algorithm was employed.

### Intraclass correlation coefficient and inter-rater reliability validation

To measure the accuracy and determine reliability of the CNN, the model’s output and analysis of the tumor ROIs were compared to the manual segmentation results from two clinical radiologists. The radiologists segmented the tumor ROI manually using freehand selection in the ImageJ software (NIH, Bethesda, MD), and values were extracted for deltaT2 analysis from the tumor ROIs. For statistical analysis, SPSS statistical software was used to calculate Intraclass Correlation Coefficient (ICC), which provides a measure of the Inter-Rater Reliability amongst various raters (neural network and radiologists) and indicates the accuracy of the model in segmenting proper tumor ROI from the negative contrast pixel field. A two-way mixed model with a confidence interval of 95% was selected, and a measure of absolute agreement was calculated with the ICC. The greater the ICC value, the more reliable the model is due to the greater degree of correlation amongst the rater’s values. In order to account for overall tumor shape, size, and ROI value, ICC was calculated in accordance to three metrics extracted individually from both the deep learning model (automatically) and by the board certified radiologists (manually) using the ImageJ software: circularity, roundness, and sum of pixel ROI (size).

### Statistical analysis

Data were presented as mean ± SD. Statistical comparisons between two groups were evaluated by the Student t test and corrected by one-way ANOVA for multiple comparisons using GraphPad Prism 5 (GraphPad Software, Inc., La Jolla, CA, USA) and IBM SPSS statistics software. A value of *p* < 0.05 was considered statistically significant.

## Supplementary information

Supplementary Legends.

Supplementary Figures.

## Data Availability

All data generated or analyzed during this study are included in this published article (and its Supplementary Information file).
