# Supplementary material for: Molecular imaging and deep learning analysis of uMUC1 expression in response to chemotherapy in an orthotopic model of ovarian cancer
Source: Sci Rep. 2020 Sep 10;10:14942. doi: 10.1038/s41598-020-71890-2 (PMC7484755; doi:10.1038/s41598-020-71890-2)
Supplement: Supplementary file 1 — Supplementary Legends. [file 41598_2020_71890_MOESM1_ESM.docx]

Supplemental Figure 1. Relative expression of uMUC1 as quantified by relative fluorescence unit (RFU) values from RT-PCR of ovarian tissue samples from tumors of mice treated with docetaxel (experimental; n = 4) and untreated mice (control; n = 5). Values are expressed as ratio compared to TBP baseline expression (* indicates p < 0.05).

Supplemental Figure 2. Corrected total cell fluorescence (CTCF) of in vitro fluorescence microscopy images of SKOV3/Luc cells after treatment with docetaxel. A. CTCF of relative uMUC1 expression indicates decreased fluorescence signal from uMUC1 in cells treated with docetaxel as compared to untreated cells. B. CTCF of Cy5.5-labeled MN-EPPT nanoparticle probe accumulation in cells treated with docetaxel compared to untreated cells. Reduced accumulation of the probe was observed in cells treated with docetaxel compared to untreated cells. (* indicates p < 0.05 from student T-test between samples).

Supplemental Figure 3. Corrected total cell fluorescence (CTCF) of ex vivo tissue histology of tumor lesions from mice treated with docetaxel (n = 5) and untreated mice (n = 3) A. CTCF of relative uMUC1 expression indicates a reduction in uMUC1 expression in mice treated with docetaxel in comparison with untreated mice. B. CTCF of Cy5.5 labeled MN-EPPT nanoparticle probe accumulation in tumor tissue of mice treated with docetaxel and untreated mice. Lower accumulation of the nanoparticle probe was observed in the treated mice as indicated by the reduced Cy5.5 CTCF (* indicates p < 0.05 from student T-test between samples).
