# Supplementary material for: Molecular imaging and deep learning analysis of uMUC1 expression in response to chemotherapy in an orthotopic model of ovarian cancer
Source: Sci Rep. 2020 Sep 10;10:14942. doi: 10.1038/s41598-020-71890-2 (PMC7484755; doi:10.1038/s41598-020-71890-2)
Supplement: Supplementary file 2 — Supplementary Figures. [file 41598_2020_71890_MOESM2_ESM.pdf]

SUPPLEMENTARY INFORMATION FOR THE MANUSCRIPT

Molecular imaging and deep learning analysis of uMUC1 expression in response to chemotherapy in an orthotopic model of ovarian cancer

Hongwei Zhao[1,2,3,4], Hasaan Hayat[1,2,5], Xiaohong Ma[1,2,6], Daguang Fan[1,2,7], Ping Wang[1,2 *], Anna Moore[1,2 *]

Affiliations:

[1] Precision Health Program, Michigan State University, East Lansing, Michigan 48823, USA.

[2] Department of Radiology, College of Human Medicine, Michigan State University, East Lansing, Michigan 48823, USA.

[3] Shanxi Medical University, Taiyuan, Shanxi, 030001, China.

[4] Department of Gynecologic Oncology, Shanxi Provincial Cancer Hospital, Taiyuan, Shanxi, 030013, China.

[5] Lyman Briggs College, Michigan State University, East Lansing, Michigan 48823, USA.

[6] Department of Radiology, National Cancer Center/Cancer Hospital, Chinese Academy of Medical Sciences and Peking Union Medical College, Beijing, 100021, China.

[7] Department of General Surgery, Shanxi People's Hospital, Taiyuan, Shanxi, 030012, China.

*Address correspondence to: Anna Moore, Precision Health Program, Department of Radiology, Michigan State University, 766 Service Road, Rm. 2022, East Lansing, MI 48823, tel. (517)-355-4091, email: moorea57@msu.edu, and Ping Wang, Precision Health Program, Department of Radiology, Michigan State University, 766 Service Road, Rm. 2020, East Lansing, MI 48823, tel. (517)-353-3817, email: wangpin4@msu.edu

Supplemental Figure 1

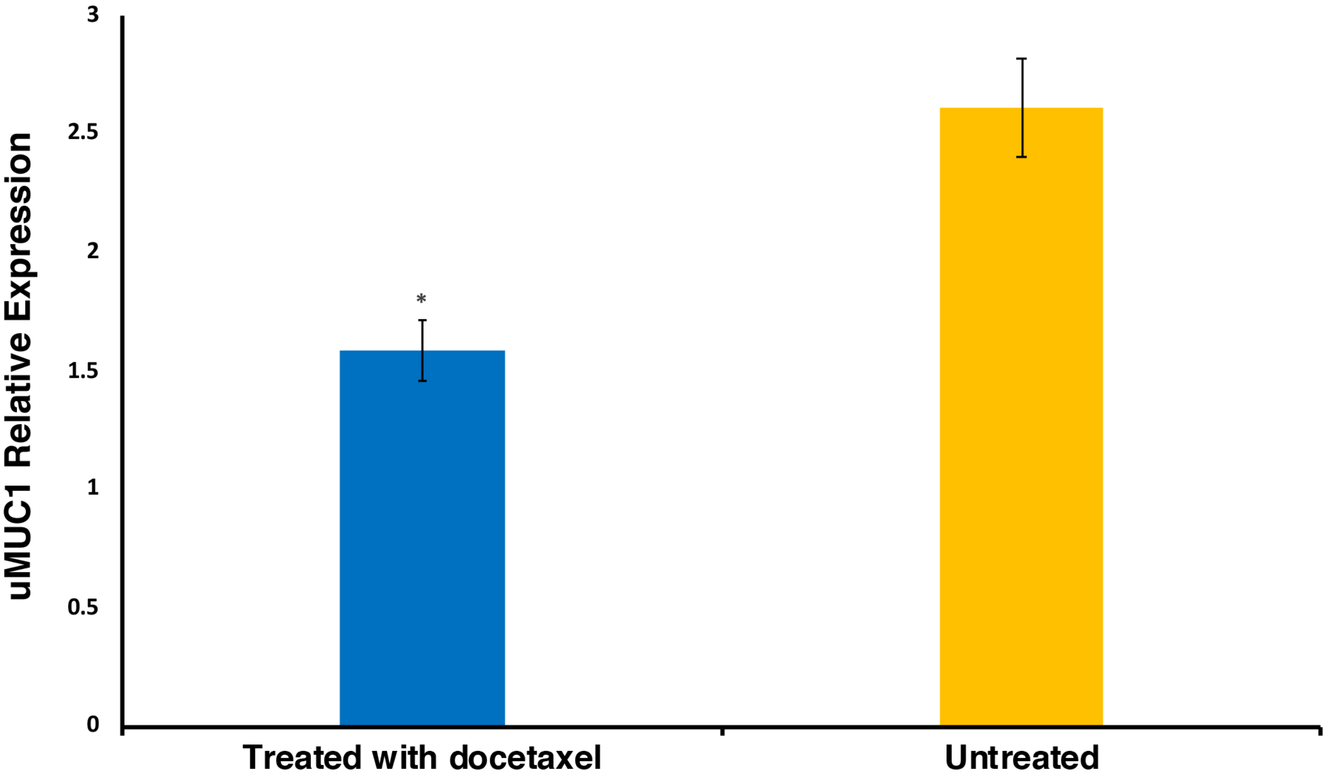

Supp Fig 2

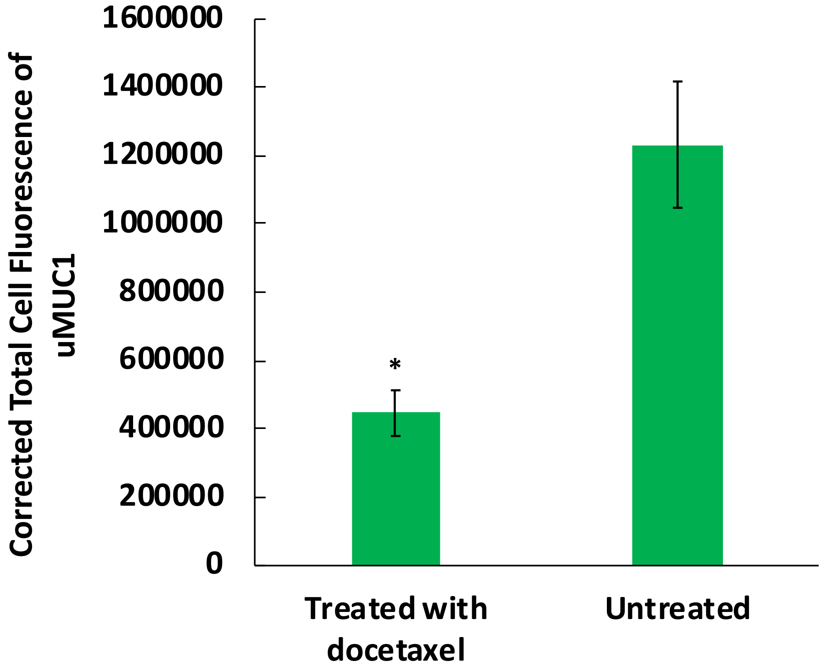

Corrected Total Cell Fluorescence of uMUC1
1600000
1400000
1200000
1000000
800000
600000
400000
200000
0
*
Treated with docetaxel
Untreated

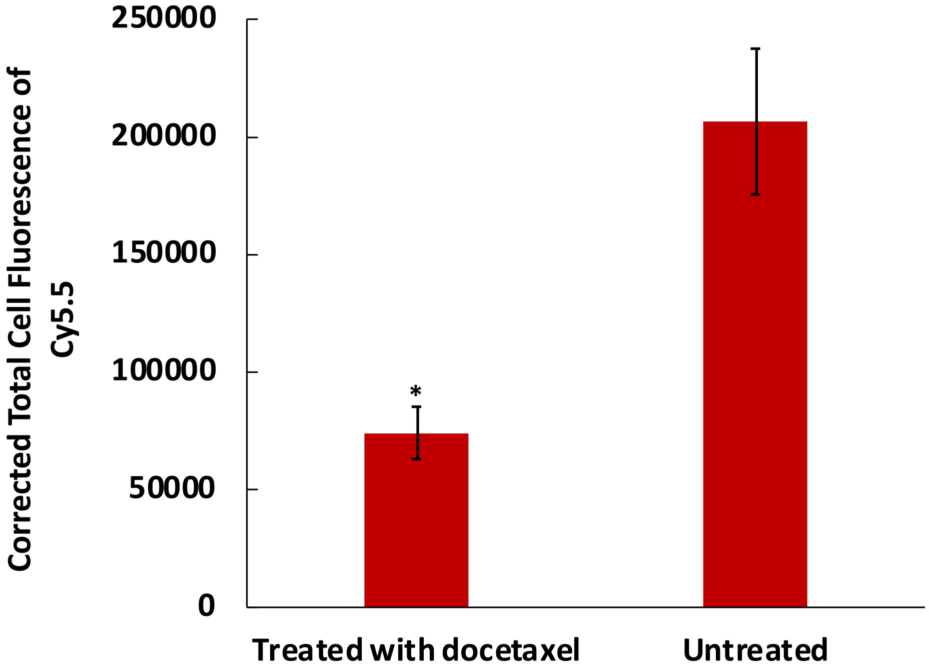

Corrected Total Cell Fluorescence of Cy5.5
250000
200000
150000
100000
50000
0
*
Treated with docetaxel
Untreated

Supp Fig 3

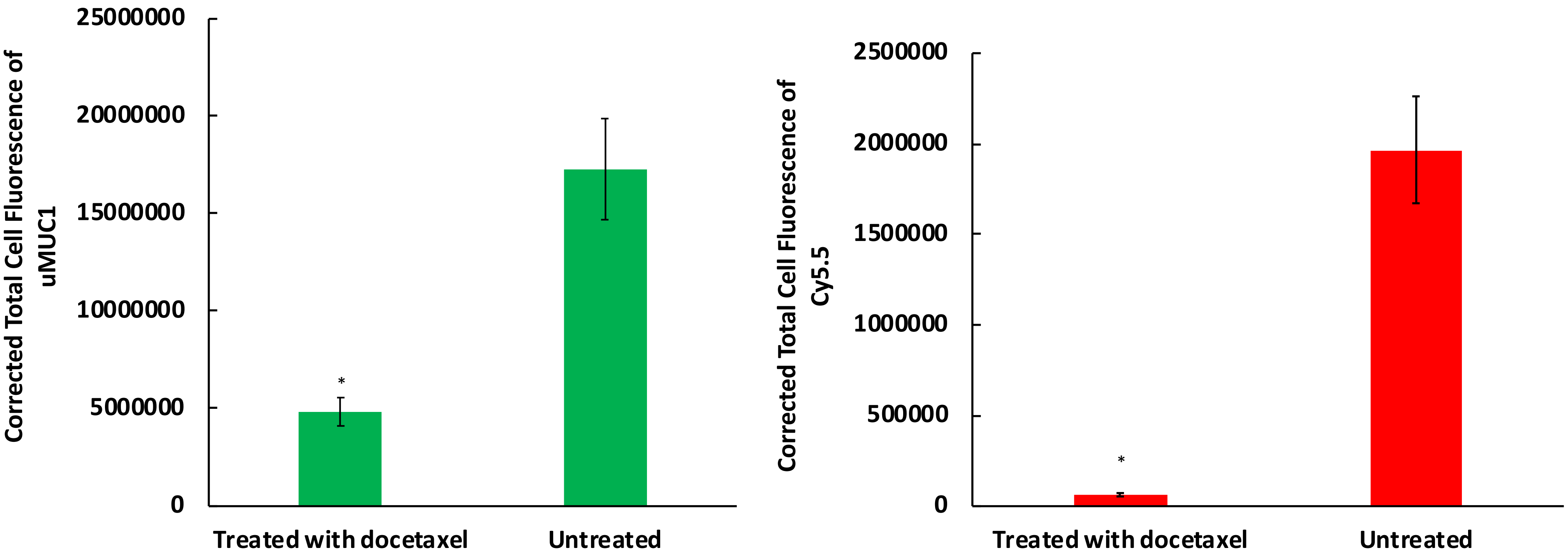

Corrected Total Cell Fluorescence of uMUC1
25000000
20000000
15000000
10000000
5000000
0
*
Treated with docetaxel
Untreated
Corrected Total Cell Fluorescence of Cy5.5
2500000
2000000
1500000
1000000
500000
0
*
Treated with docetaxel
Untreated
